# Two parallel pathways for ferric and ferrous iron acquisition support growth and virulence of the intracellular pathogen *Francisella tularensis* Schu S4

**DOI:** 10.1002/mbo3.342

**Published:** 2016-02-25

**Authors:** Natalie Pérez, Richard Johnson, Bhaswati Sen, Girija Ramakrishnan

**Affiliations:** ^1^ Department of MedicineDivision of Infectious Diseases and International HealthUniversity of VirginiaCharlottesvilleVirginia22908

**Keywords:** Copper, FeoB, gallium, membrane transport, siderophore.

## Abstract

Iron acquisition mechanisms in *Francisella tularensis*, the causative agent of tularemia, include the *Francisella* siderophore locus (*fsl)* siderophore operon and a ferrous iron–transport system comprising outer‐membrane protein FupA and inner‐membrane transporter FeoB. To characterize these mechanisms and to identify any additional iron uptake systems in the virulent subspecies *tularensis*, single and double deletions were generated in the *fsl* and *feo* iron acquisition systems of the strain Schu S4. Deletion of the entire *fsl* operon caused loss of siderophore production that could be restored by complementation with the biosynthetic genes *fslA* and *fslC* and Major Facilitator Superfamily (MFS) transporter gene *fslB*. ^55^Fe‐transport assays demonstrated that siderophore‐iron uptake required the receptor FslE and MFS transporter FslD. A Δ*feoB*′ mutation resulted in loss of ability to transport ferrous iron (^55^Fe^2+^). A Δ*feoB*′ Δ*fslA* mutant that required added exogenous siderophore for growth in vitro was unable to grow within tissue culture cells and was avirulent in mice, indicating that no compensatory cryptic iron uptake systems were induced in vivo. These studies demonstrate that the *fsl* and *feo* pathways function independently and operate in parallel to effectively support virulence of *F. tularensis*.

## Introduction

Iron is fundamental to basic metabolic processes and homeostasis of every domain of life; nevertheless, aerobic organisms need to tightly regulate levels of free iron in order to limit the formation of damaging reactive oxygen species (Freinbichler et al. 2011). Microbial pathogens, therefore, face iron limitation within the mammalian host where the majority of iron is sequestered; additional iron restriction results from mechanisms that are activated in response to perceived infection including an increase in levels of lipocalin to bind up bacterial siderophores, transferrin to scavenge free iron, and the hormone hepcidin, which influences systemic iron metabolic pathways (Nairz et al., 2010).

Iron in living systems transitions between two forms that have intrinsically different properties: the reduced ferrous form (Fe^2+^) is highly soluble and is thought to be the primary constituent of the cytoplasmic labile iron pool, while the relatively insoluble oxidized ferric form (Fe^3+^) is believed to mainly be contained within protein complexes (Diouhy and C. E 2013; Hider and Kong 2013). Bacterial pathogens, therefore, need different mechanisms to access these two forms of iron.

*Francisella tularensis*, the etiological agent of tularemia, is a Gram‐negative bacterium with a very low infectious dose (<10 CFU) (Jones et al. 2012; Celli and Zahrt 2013). It is commonly transmitted by arthropod vectors in the wild and can infect a wide range of mammalian hosts. The bacterium is internalized by and replicates within the cytoplasm of a variety of host cells, most notably macrophages and hepatocytes. The high virulence and rapid intracellular growth of this pathogen implies that it possesses mechanisms to efficiently acquire essential nutrients from the host (Abu Kwaik and Bumann 2013).

The importance of iron for intracellular growth of *F. tularensis* was first demonstrated using a macrophage infection model where bacterial replication was inhibited by deferoxamine treatment and iron acquired by the macrophage through transferrin or nontransferrin‐dependent mechanisms was able to support bacterial growth (Fortier et al. 1995). Several studies have demonstrated that *F. tularensis* encodes separate mechanisms for ferrous and ferric iron acquisition to support its pathogenic lifestyle (Deng et al. 2006; Sullivan et al. 2006; Lindgren et al. 2009; Ramakrishnan et al. 2012; Thomas‐Charles et al. 2013).

The three subspecies of *F. tularensis*:* tularensis*,* holarctica* and *mediasiatica* display differences in virulence, and there is great interest in discerning the mechanisms that contribute to these differences. Variations in iron metabolism appear to influence the physiology of the subspecies; the *holarctica* strains have higher levels of bacterioferritin and increased internal iron stores relative to *tularensis* leading to differences in susceptibility to hydrogen peroxide–induced killing (Hubálek et al., 2004; Lindgren et al., 2011). Differences in mechanisms of iron acquisition could potentially contribute to these variations.

**Figure 1:**
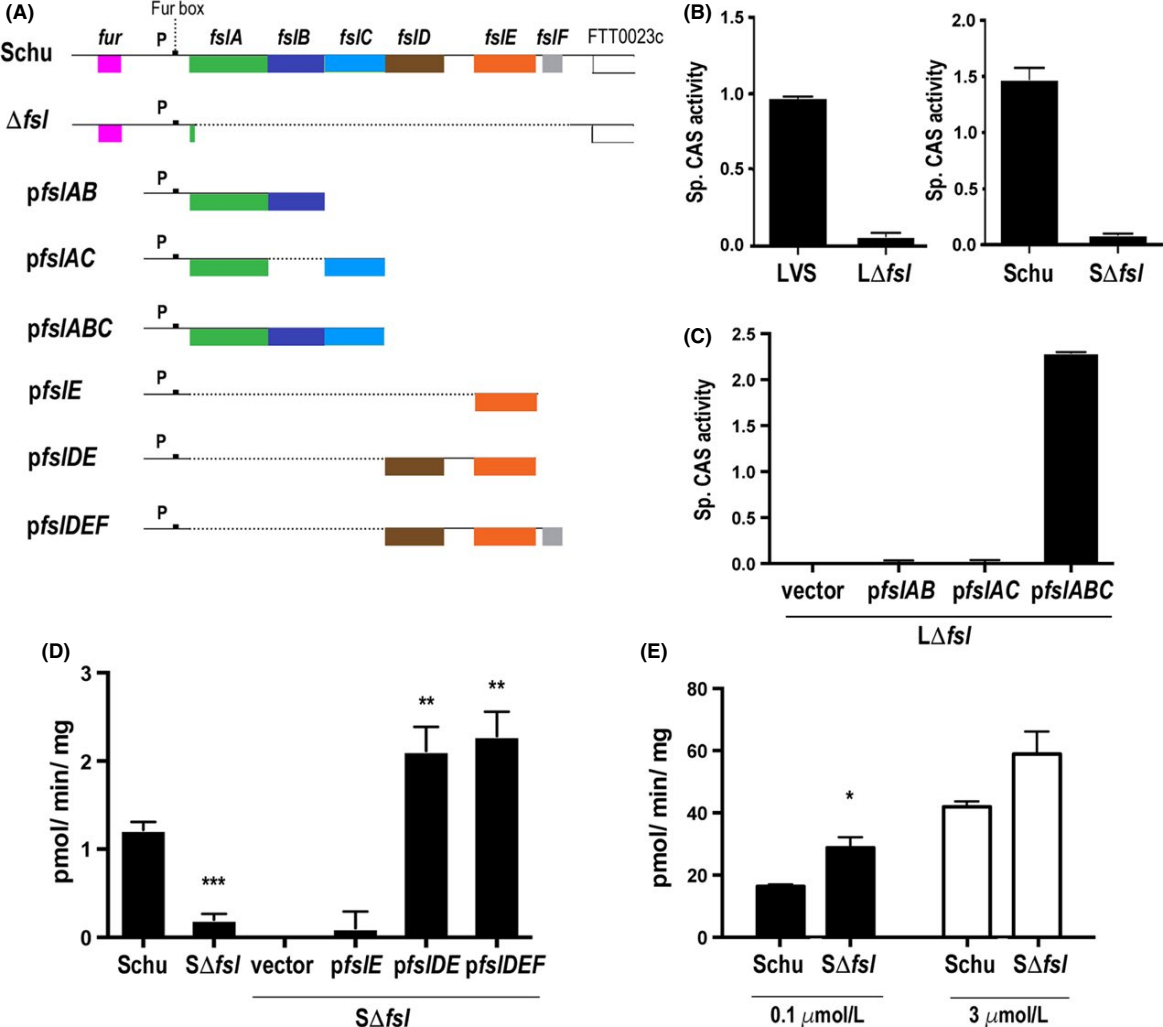
Identification of *fsl* genes involved in siderophore production and uptake. (A) Schematic of *fsl* operon and complementing plasmids. The *fsl* operon of Schu S4 is shown on top and corresponding region of the Δ*fsl* mutant is shown underneath. The sequences deleted in the mutant are depicted by the dotted line. P indicates the *fsl* promoter. The different complementing plasmids are shown with the *fsl* genes that they carry. (B,C) Siderophore production in different strains determined by Chrome Azurol S (CAS) activity of culture supernatants. LΔ*fsl* and SΔ*fsl* represent the Δ*fsl* mutants of live vaccine strain (LVS) and Schu S4, respectively. (B) Siderophore production in Δ*fsl* mutants of LVS and Schu S4. (C) Complementation of the LVS Δ*fsl* strain with plasmids to restore siderophore production. (D) Siderophore‐mediated uptake of ^55^Fe^3+^ in Schu Δ*fsl* mutant and complemented strains. Siderophore‐^55^Fe complexes were added to iron‐starved cultures and the rates of ^55^Fe uptake were determined. (E) Ferrous iron uptake by Schu Δ*fsl* mutant. The rate of uptake of 0.1 or 3.0 *μ*mol/L ^55^Fe^2+^ by iron‐starved Schu S4 and Δ*fsl* mutant were determined in the presence of ascorbate. For (B–E) values are expressed as mean ± standard error of the mean (SEM); assays were carried out in triplicate for B and C and in quadruplicate for D and E. Experiments were repeated two or more times with similar results. Results were analyzed with two‐tailed Student's *t*‐test. Mutants were compared with parent strain, and complemented strains were compared with the vector‐transformed controls. *P = 0.02, **P = 0.002, ***P ≤ 0.0001.

Many bacteria produce siderophore molecules that chelate ferric iron in the environment for subsequent uptake by dedicated transport systems (Miethke and Marahiel 2007). Strains of *F. tularensis* and the related *Francisella novicida* species secrete a polycarboxylate siderophore similar to rhizoferrin, and siderophore production is governed by the conserved *Francisella* siderophore locus (*fsl)* (*frg*) operon under control of the Fur repressor (Deng et al. 2006; Sullivan et al. 2006; Buchan et al. 2008; Ramakrishnan et al. 2008) (Fig. 1A represents the chromosomal *fsl* locus in the *tularensis* subspecies strain Schu S4). The *fslA* and *fslC* genes encode a siderophore synthetase and a decarboxylase, respectively, required for *Francisella* rhizoferrin production (Deng et al. 2006; Sullivan et al. 2006; Lindgren et al. 2009; Thomas‐Charles et al. 2013). *fslB* and *fslD* encode putative inner‐membrane proteins belonging to the Major Facilitator Superfamily (MFS). Mutation in *fslB* in the *F. novicida* strain U112 led to diminished production of siderophore (Kiss et al. 2008). *fslD* function in *Francisella* has not been explored.

The *Francisella* rhizoferrin receptor FslE, which bears no significant sequence similarity to known siderophore receptors, is also encoded by the *fsl* operon (Milne et al. 2007; Kiss et al. 2008; Ramakrishnan et al. 2008). Transport of the siderophore–iron complex across the outer membrane of Gram‐negative bacteria is typically powered by the TonB–ExbB–ExbD complex in the inner membrane (Miethke and Marahiel 2007; Chu et al. 2010). The *F. tularensis* genome lacks the genes for TonB, ExbB, and ExbD, and therefore, siderophore‐mediated iron uptake in this Gram‐negative organism is atypical. The last gene of the operon, *fslF,* shows sequence differences between the *F. tularensis* and *F. novicida* strains; *fslF*_Schu S4_ encodes a 114‐amino acid protein predicted to be in the inner membrane, while FslF_U112_ is a larger protein that includes sequences of an independent ORF downstream in *F. tularensis* (FTT0023c in Fig. 1A). Whether FslF retains a siderophore‐associated function in the different strains is therefore of question.

Dedicated outer and inner‐membrane proteins for transport of ferrous iron have been identified in *Francisella* species. The outer‐membrane protein FupA, a paralog of the siderophore receptor FslE, is also associated with iron metabolism (Lindgren et al. 2009) and mediates high‐affinity transport of ferrous iron (Ramakrishnan et al. 2012). The inner‐membrane ferrous iron transporter FeoB is encoded by all sequenced *Francisella* genomes and has been functionally characterized in the live vaccine strain (LVS) of the *F. tularensis holarctica* subspecies (Thomas‐Charles et al. 2013; Pérez and Ramakrishnan 2014).

Transcriptional profiling of Schu S4‐infected mouse bone marrow derived macrophages showed that genes within the *fsl* were upregulated during the course of infection (Wehrly et al. 2009). Screens of transposon mutant libraries and testing of individual mutants indicated that the *fsl* genes and the ferrous iron transporter gene *feoB* are required for optimal virulence of *F. novicida* and of LVS (Su et al. 2007; Weiss et al. 2007; Thomas‐Charles et al. 2013; Pérez and Ramakrishnan 2014). Deletion of *fupA* in Schu S4 led to partial attenuation in mice (Twine et al. 2005; Ramakrishnan et al. 2012), while a mutant lacking both FslE and FupA remained avirulent for mice even with 1000 LD_50_ delivered by the subcutaneous route (Ramakrishnan et al. 2012). Screens with mutants of U112 and Schu S4 have suggested that additional genes could be involved in iron acquisition, but their roles have not been definitively characterized (Crosa et al. 2009; Lindgren et al. 2015).

We previously showed using in vitro and intracellular growth assays and a mouse infection model that the *Francisella* rhizoferrin and *feoB*‐dependent transport mechanisms were the only operational iron acquisition systems in LVS (Pérez and Ramakrishnan 2014). An important cause of attenuation in LVS is the genomic recombination event that generated a hybrid gene between *fupA* and the downstream gene *fupB* (Salomonsson et al. 2009). The FupA paralog of LVS FupA/B is less efficient at ferrous iron uptake; in addition, FslE_LVS_ is less efficient than FslE_Schu S4_ and FupA/B contributes to siderophore‐mediated iron uptake in LVS (Sen et al. 2010; Ramakrishnan and Sen 2014). These findings highlight differences in iron uptake capability of LVS and Schu S4.

To gain a comprehensive understanding of iron acquisition in the virulent *tularensis* subspecies, we have queried gene function and defined attributes of iron uptake in Schu S4. We determined that the pathways for *Francisella* rhizoferrin‐mediated ferric iron acquisition and FupA–FeoB‐dependent ferrous iron uptake are parallel and independent, and that in conjunction, these two systems satisfy the iron requirements for growth and full virulence of the organism.

## Results

### The *fsl* operon encodes requisite functions for siderophore biosynthesis, transport, and siderophore‐dependent iron acquisition

We generated mutants lacking the entire *fsl* operon (*fslA*‐*fslF*) (Fig. 1A) in both Schu S4 (SΔ*fsl*) and LVS (LΔ*fsl*). As might be predicted, deletion of the entire *fsl* locus resulted in loss of siderophore production as determined by the Chrome Azurol S (CAS) assay (Fig. 1B). We then introduced combinations of *fsl* genes back into the Δ*fsl* mutants and tested for restoration of siderophore‐associated function. Specifically, we reintroduced biosynthetic gene *fslA* in combination either with *fslB*,* fslC* or *fslB + fslC* back into the LΔ*fsl* mutant and assessed siderophore production. As shown in Figure 1C, *fslA, fslB,* and *fslC* were all required and were sufficient to restore siderophore production. This is consistent with previous findings that mutants in the individual genes are deficient in siderophore production (Deng et al. 2006; Sullivan et al. 2006; Kiss et al. 2008; Thomas‐Charles et al. 2013). We conclude that siderophore synthesized by action of the FslA and FslC enzymes is transported out of the cytoplasm by the MFS transporter FslB.

The Δ*fsl* mutants are predicted to be deficient at siderophore utilization and the SΔ*fsl* mutant showed loss of siderophore‐mediated ^55^Fe uptake capability (Fig. 1D). Expression of the siderophore receptor gene *fslE* alone was not sufficient to restore siderophore uptake capability to the mutant, but introduction of *fslD* along with *fslE* did restore this function. These results indicated that the outer‐membrane receptor FslE and the inner‐membrane MFS transporter FslD are involved in siderophore‐iron uptake.

Introduction of *fslF* with *fslD* and *fslE* did not further augment siderophore‐mediated ^55^Fe uptake. This complementation assay could potentially mask a subtle involvement of *fslF* in iron uptake, and we, therefore, generated a Schu Δ*fslF* strain lacking only the last gene of the operon. The Δ*fsl*F mutant, however, showed no differences relative to the parent strain in assays for siderophore production and for siderophore‐mediated uptake (data not shown), suggesting that FslF does not contribute to siderophore utilization in *F. tularensis*.

Although deficient in siderophore‐mediated ^55^Fe uptake, the SΔ*fsl* mutant was capable of ferrous iron (^55^Fe^2+^) acquisition (Fig. 1E); in fact, the mutant strain was more proficient than the parent strain in ferrous iron uptake, with the difference being statistically significant at the lower iron concentrations (100 nmol/L). This suggests that ferrous iron uptake is deregulated in the mutant to compensate for loss of siderophore function.

In summary, complementation studies with the Δ*fsl* mutants definitively established that the *fslA*,* fslB,* and *fslC* genes were responsible for siderophore production while *fslD* and *fslE* were essential for siderophore‐mediated iron uptake. Additionally, these studies demonstrated clearly that ferrous iron uptake functions independently of the *fsl* siderophore operon.

### FeoB is necessary for optimal growth under iron limitation

Previous studies indicated that inner‐membrane ferrous iron transport in LVS is primarily mediated by FeoB (Pérez and Ramakrishnan 2014). To determine the importance of FeoB function in Schu S4, we generated a deletion mutant predicted to express a truncated FeoB protein lacking the carboxy‐terminal half (Δ*feoB*′).

**Figure 2:**
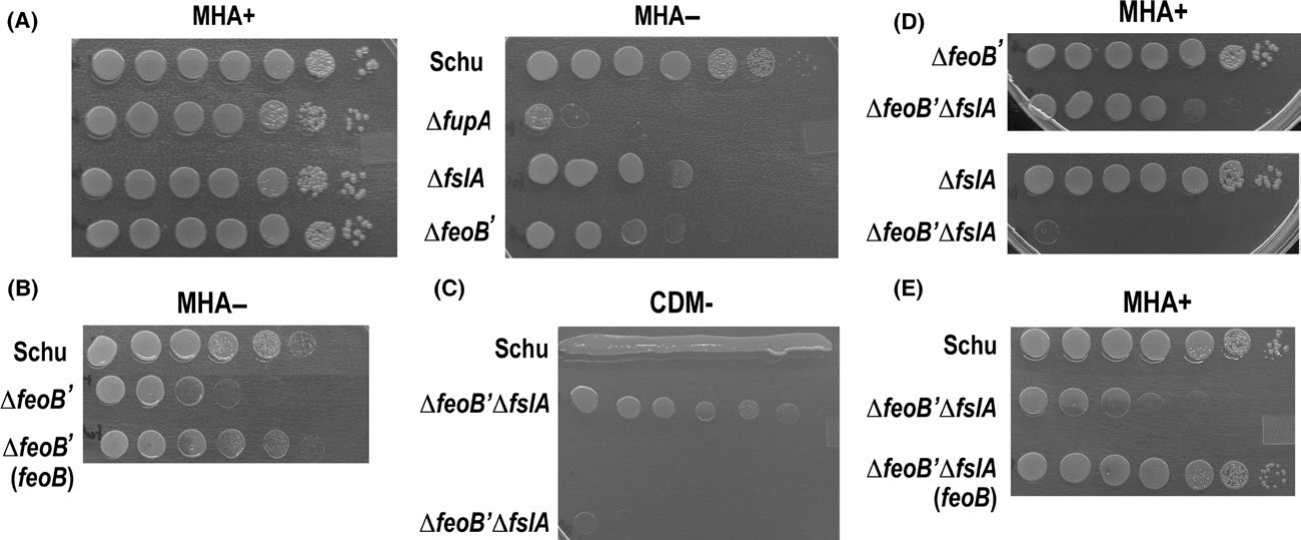
Growth of Schu S4, iron acquisition mutants and complemented strains on iron‐replete or iron‐limiting agar. Except for the ΔfeoB' ΔfslA mutant, all strains were grown overnight in iron‐replete che‐CDM, washed and resuspended to an OD595 of 1.0. Ten‐ fold serially diluted suspensions were spotted on iron‐replete MHA+ (A,D,E) or iron‐limiting MHA‐ (A,B) and grown for 3 days at 37oC before recording the images. (C‐E) The ΔfeoB' ΔfslA mutant was grown on MHA+ supplemented with siderophore and bacteria were scraped into che‐CDM and washed before serial dilutions were spotted on the agar plates as indicated. In C, two sets of dilutions of the ΔfeoB' ΔfslA were spotted on the iron‐limiting CDM‐ plate, and Schu S4 bacteria were streaked in the vicinity of one set of dilutions.

Growth of the Schu Δ*feoB*′ mutant was compared to the parent Schu S4 and to Δ*fupA* and Δ*fslA* mutants known to be important for ferrous iron acquisition and siderophore biosynthesis, respectively (Sullivan et al. 2006; Lindgren et al. 2009; Ramakrishnan et al. 2012). Tenfold serial dilutions of the bacteria were spotted on iron‐replete Mueller–Hinton agar (MHA)+ or on iron‐limiting MHA−. While the different strains grew similarly on MHA+, there was a dramatic difference on MHA− (Fig. 2A). Schu S4 was able to grow to 10^−6^ dilution on MHA−, while the mutants showed different levels of reduction in growth. The Δ*fslA* mutant grew to 10^−4^ dilution and the Δ*feoB*′ and the Δ*fupA* mutant grew less well. The Δ*fupA* mutant had a more severe growth defect than the Δ*feoB*′ mutant, suggesting that FupA may have additional functions besides that of ferrous iron acquisition. The Δ*feoB*′ mutant complemented with a wild‐type copy of *feoB* in *cis* was able to grow like the wild‐type Schu S4 on MHA− plates (Fig. 2B). Taken together, these results indicated that the ferric‐siderophore system and FeoB‐mediated ferrous iron acquisition are both required for normal growth on iron‐limiting MHA plates.

### FeoB is essential for transport of ^55^Fe^2+^ by Schu S4

**Figure 3:**
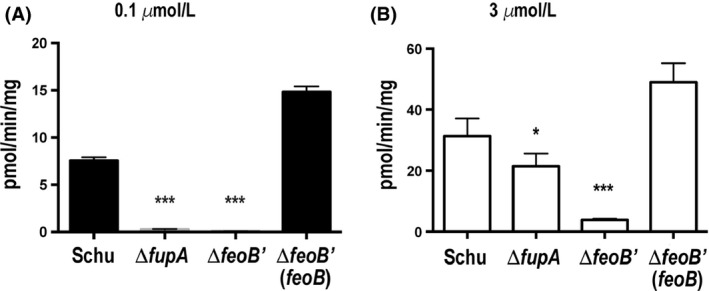
Uptake of ^55^Fe^2+^ by Schu S4 and iron acquisition mutants. Iron‐starved cultures of Schu S4, the Δ*fupA* and Δ*feoB*′ mutants and the complemented Δ*feoB*′ mutant were assayed for the ability to take up ^55^Fe^2+^ when present at 0.1 *μ*mol/L (A) or 3 *μ*mol/L (B) concentration. Values are expressed as means + standard error of the mean (SEM) of quadruplicate assays. Values were compared to those of Schu S4. *P < 0.05, ***P ≤ 0.0001.

We previously showed that the outer‐membrane protein FupA is associated with high‐affinity uptake of ferrous iron (Fe^2+^), important at limiting iron concentrations (Ramakrishnan et al. 2012). We compared ^55^Fe^2+^ uptake in the Δ*feoB*′ mutant, the Δ*fupA* mutant, and parent Schu S4. As expected, the Δ*fupA* mutant was deficient at iron transport particularly at the lower ferrous iron concentration (0.1 *μ*mol/L), but the Δ*feoB*′ mutant was defective for uptake of ferrous iron both at the low and at the high 3.0 *μ*mol/L iron concentration (Fig. 3). This uptake defect was abrogated upon complementation with a wild‐type copy of *feoB*. These results indicate that whereas the outer membrane has low‐affinity channels in addition to the high‐affinity FupA transporter, FeoB is the only functional inner‐membrane transporter for ferrous iron in Schu S4.

### Schu *ΔfeoB*′ mutant shows increased expression of the *fsl* operon

**Figure 4:**
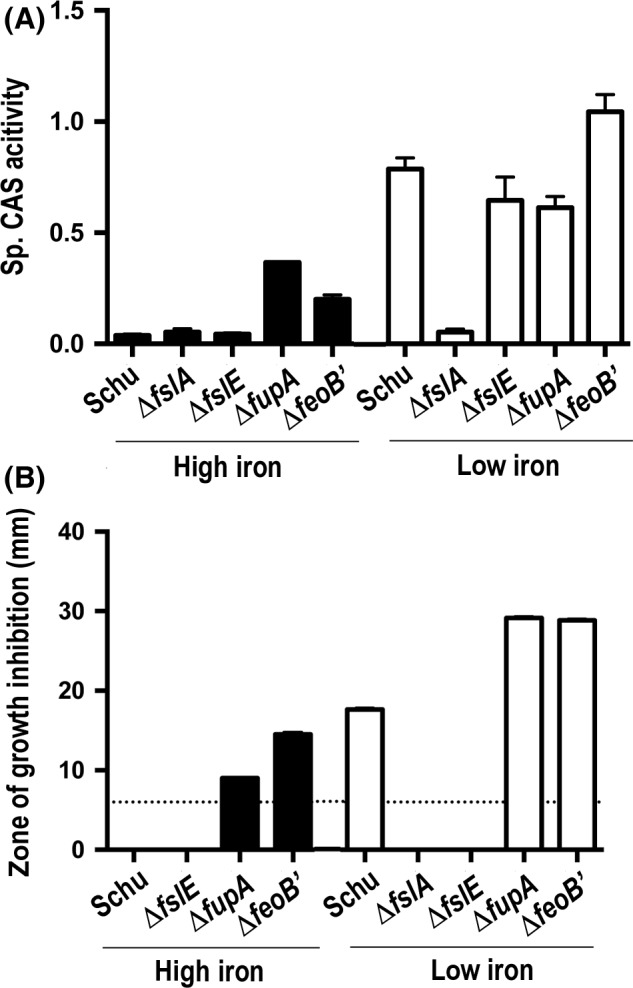
Tests for siderophore activity in iron acquisition mutants. (A) Siderophore production as measured by Chrome Azurol S (CAS) activity. Cultures of Schu S4 and the iron acquisition mutants were grown to log phase in che‐CDM made replete (high iron) or limiting (low iron) with ferric pyrophosphate (FePPi) supplementation. The CAS activity was determined in triplicate assays and the means + standard error of the mean (SEM) are shown. (B) Gallium sensitivity assays on agar. Bacteria from exponential phase cultures were resuspended in Chamberlin's defined medium (CDM) to an OD_595_ of 1 and spread on Mueller–Hinton agar (MHA)+ (high iron) or MHA− (low iron) plates. 10 *μ*L of 50 mmol/L gallium nitrate was spotted on paper discs placed on the seeded plates. Growth was scored after one MHA+ and two MHA− days at 37°C and the diameter of the zone of growth inhibition, if present, was measured. Assays were carried out in triplicate and results are presented as means and standard error of the mean. The dotted line at 6 mm represents the diameter of the paper discs.

A Δ*fslA* mutant is unable to produce siderophore while a Δ*fupA* mutant secretes siderophore in both iron‐replete and iron‐limiting conditions (Sullivan et al. 2006; Lindgren et al. 2009). Using the CAS assay with cultures grown in liquid Chamberlin's defined medium (CDM), we found that siderophore production is also deregulated in the Schu S4 Δ*feoB*′ mutant under iron‐replete (high iron) conditions, similar to the Δ*fupA* mutant (Fig. 4A).

Siderophores can bind gallium in a manner similar to ferric iron and gallium‐siderophore complexes are recognized and taken up by microbial siderophore‐transport systems (Emery and Hoffer 1980). Gallium is toxic to bacteria, and siderophore‐mediated uptake of gallium leads to bacterial growth inhibition (Carrano et al., 1996a, 1996b). We evaluated siderophore activity in the *F. tularensis* strains by testing for growth sensitivity in the presence of gallium. We first seeded the different strains on iron‐rich MHA+ or iron‐limiting MHA− plates and spotted gallium nitrate on 6 mm paper discs, and then examined subsequent growth of the lawn. The siderophore is normally only expressed under iron limitation, and as expected, Schu S4 showed a zone of growth inhibition around the gallium discs only on MHA− and not on MHA+ plates (Fig. 4B). The Δ*fslA* and Δ*fslE* mutants showed no zone of inhibition on either plate confirming that an intact *fsl* siderophore uptake system is required for gallium toxicity. The *fupA* and the *feoB* mutants, in contrast, showed zones of growth inhibition around the paper discs on MHA+ and even larger zones on MHA− plates compared to Schu S4. These results confirmed that siderophore‐mediated iron uptake is deregulated in the *fupA* and *feoB* mutants and suggested that loss of ferrous iron uptake capability shifts the reliance of the bacterial growth to the siderophore system.

### A Schu *ΔfeoB*′ *ΔfslA* mutant relies on exogenous siderophore for growth

We generated a Δ*feoB*′ Δ*fslA* mutant to test if Schu S4 possesses iron uptake mechanisms separate from *fsl* siderophore and *feoB* encoded systems. As shown in Figure 2C, serial dilutions of the double mutant could grow in the vicinity of, but not distant from Schu S4 on iron‐limiting CDM agar (CDM−). However, even in iron‐replete conditions (MHA+), the double mutant could only grow adjacent to a siderophore producer (Schu S4 or Schu Δ*feoB*′), but not the siderophore‐deficient Δ*fslA* mutant (Fig. 2D, 2E). Complementation with *feoB* restored its ability to grow as well as the parent strain on MHA+ (Fig. 2E). These data made it clear that virulent Schu S4, like LVS, has only the two iron uptake mechanisms of *fsl* and *feoB* for growth in vitro. Since supplementation with purified *Francisella* rhizoferrin was required for routine maintenance of this strain, all further experiments with this strain were conducted with bacteria grown on siderophore‐containing MHA+ plates.

### *feoB* and *fslA* functions support intracellular growth of Schu S4

**Figure 5:**
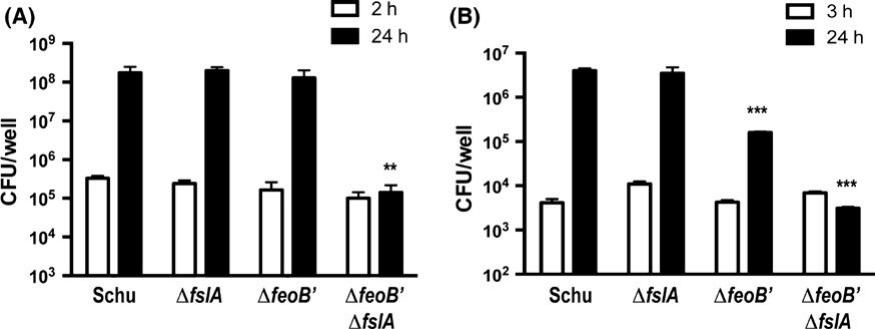
Intracellular growth of Schu S4 and mutants. Tissue culture cells seeded in 24 well plates were infected with bacteria at multiplicity of infection (MOI) as stated below. Bacterial entry at 2 h and replication over a 24 h period were determined by lysis and plating on Mueller–Hinton agar (MHA)+ agar to determine bacterial load. Purified *F. tularensis* siderophore was topically applied to MHA+ plates for growth of the Δ*feoB*′ Δ*fslA* mutant. Values are expressed as the CFU means from quadruplicate wells ± standard error of the mean (SEM). Significance calculations were made relative to Schu S4. **P < 0.01, ***P < 0.0005. (A) J774A.1 infected at a MOI 15–25 and (B) Human liver carcinoma cell line (HepG2) infected at a MOI of 80–140.

The Schu S4 iron acquisition mutants were tested for their ability to enter and replicate within cell lines of different lineage, J774A.1 and human liver carcinoma cell line (HepG2) (Fig. 5). In the murine macrophage J774A.1 cell line, the single deletion mutants of Schu S4, Δ*fslA,* and Δ*feoB*′ were able to replicate to numbers comparable to wild type 24 h post infection (Fig. 5A). This implies that both ferrous and ferric iron sources are available to the intracellular pathogen in this cell type. The *fslA* mutant was able to grow to levels comparable to wild‐type Schu S4 also in the human hepatocyte cell line, HepG2 (Fig. 5B). The *feoB*′ mutant, however, showed a reduced ability to replicate within this cell line suggesting that levels of ferric iron are limiting and that ferrous iron is the more available iron source in this cell line. The Schu S4 Δ*feoB*′ Δ*fslA* mutant was unable to grow in either the J774A.1 or HepG2 strains. This defect for intracellular growth indicates that *fsl* and *feo* encode the only iron acquisition systems for growth in the intracellular environment of these cell types.

### The Schu *ΔfeoB*′ *ΔfslA* mutant is avirulent in mice

**Figure 6:**
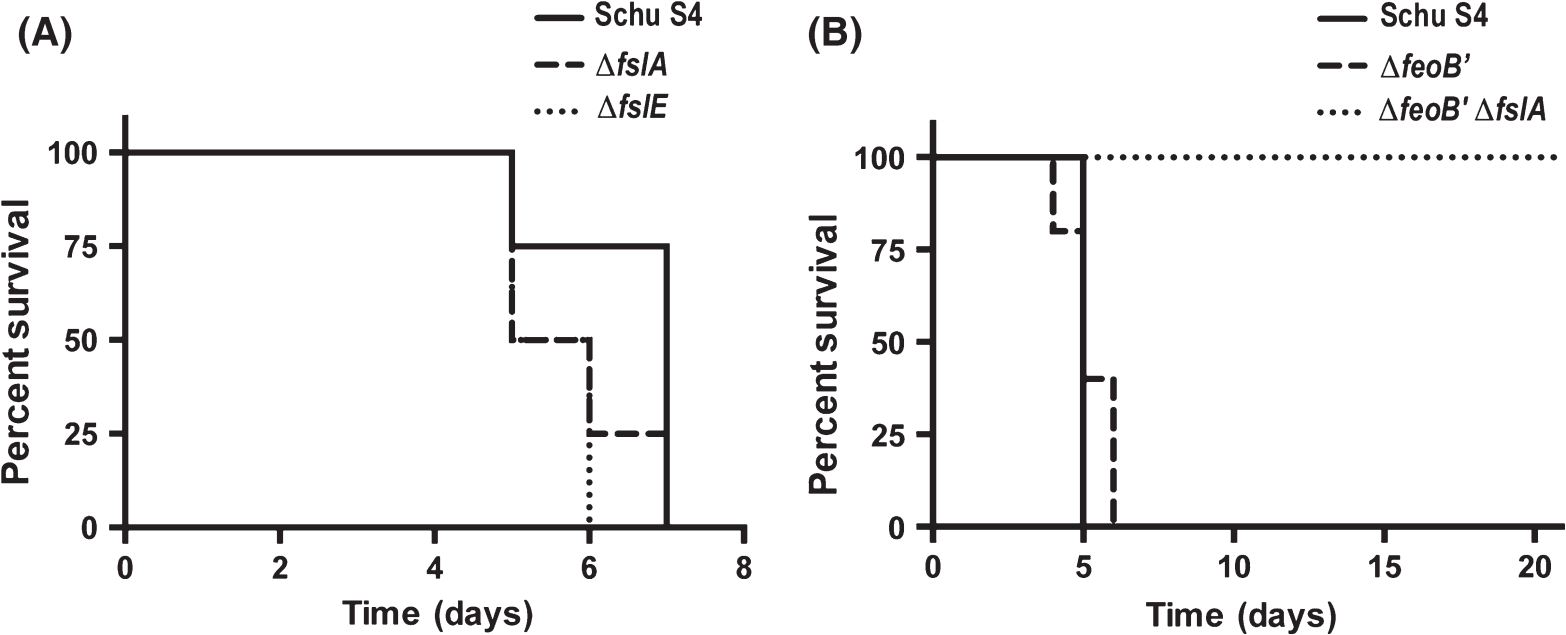
Virulence of Schu S4 and iron acquisition mutants in mice. 6–8 week old C57/BL6 mice were subcutaneously infected with ~25 CFU of the different strains. Actual CFUs delivered were determined by plating on Mueller–Hinton agar (MHA)+, with additional siderophore supplementation for growth of the Δ*feoB*′ Δ*fslA* mutant. Groups of 4 mice were used in (A) while 5 mice were used in (B) Survival curves were not significantly different among the single mutants. All mice survived infection with the Δ*feoB*′ Δ*fslA* mutant. Survivors did not succumb when subsequently challenged with 25 CFU Schu S4.

A small dose of <10 CFU Schu S4 is sufficient to kill C57BL/6 mice by any route of infection. We infected mice by the subcutaneous route with approximately 25 CFU of wild‐type Schu S4 or the different iron acquisition mutants to evaluate the importance of these systems for virulence. Mice infected with Schu S4 all died between 5 and 7 days postinfection. The single mutants in siderophore acquisition genes (Δ*fslA*, Δ*fslE*) showed no reduction in time to death of the infected mice (Fig. 6A). Mice infected with the Δ*feoB*′ mutant also died within the same time frame (Fig. 6B). The Schu Δ*feoB*′ Δ*fslA* strain, however, lived beyond 21 days post infection. These surviving mice were subsequently challenged with 25 CFU Schu S4 and monitored for 14 days more. Four of the five mice previously infected with the Δ*feoB*′ Δ*fslA* mutant survived lethal Schu S4 challenge. In an additional experiment, we attempted to calculate the LD_50_ of the Schu S4 Δ*feoB*′ Δ*fslA* mutant with increasing doses and found that even 25,000 CFU did not kill a mouse. Results with the Schu Δ*feoB*′ Δ*fslA* mutant resemble those seen with the Δ*fupA* Δ*fslE* mutant which is also highly attenuated for virulence, and is similarly able to protect mice from subsequent challenge with Schu S4 (Ramakrishnan et al. 2012). Overall, these experiments confirm that no cryptic pathways for iron acquisition are induced and that the *fsl* and *feo* pathways satisfy the iron requirements to support full virulence of Schu S4 in mice.

### FupA promotes transport of copper in addition to ferrous iron

**Figure 7:**
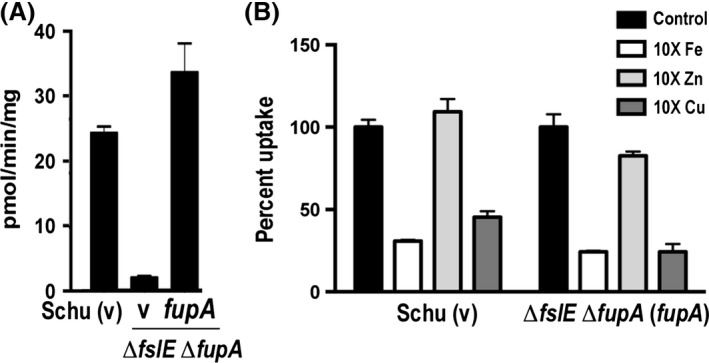
High‐affinity uptake of ^55^Fe^2+^ in the presence of competing metal ions. High‐affinity ferrous iron transport using 0.1 *μ*mol/L ^55^Fe^2+^ was studied in Schu S4 and a *fupA* complemented Δ*fslE* Δ*fupA* mutant as described in the legend to Figure 1E. (A) High‐affinity transport of ferrous iron by Schu S4 is dependent on FupA. Uptake of 0.1 *μ*mol/L ^55^Fe^2+^ is only restored to a Δ*fslE* Δ*fupA* mutant when it is complemented by a wild‐type copy of *fupA* and not by vector (v) alone. (B) ^55^Fe^2+^ uptake in the presence of competing metal ions. Reactions contained a 10‐fold molar excess of nonradioactive metal ions added as FeCl_3_, CuCl_2_, or ZnCl_2_. Rates of uptake are represented as mean ± standard error of the mean (SEM) of assays done in triplicate. Experiments were repeated at least once more with similar results.

Although ^55^Fe uptake experiments indicated that *fupA* and *feoB* are both involved in ferrous iron uptake, mutants in these genes demonstrated different growth phenotypes; the Δ*fupA* mutant that is affected in high‐affinity ferrous iron uptake had a more severe growth defect on iron‐limiting agar than the Δ*feoB*′ mutant that has lost all ability to transport ferrous iron (Fig. 2A). Although an insertion mutation in *fupA* was found to cause membrane destabilization in *F*. *novicida* (Nallaparaju et al. 2011), we have not observed such effects in the Schu Δ*fupA* mutant. We considered the possibility that FupA mediates transport of substrates besides iron and tested the ability of other metals to compete with high‐affinity transport of ^55^Fe^2+^ in Schu S4 (data not shown). The presence of ascorbate in the transport assay buffer ensures that the metals ions stay in the reduced form. In these preliminary experiments, we determined that transport of 0.1 *μ*mol/L ^55^Fe^2+^ by Schu S4 could be inhibited in the presence of an excess of nonradioactive Fe or Cu salts. To discern the specific role of FupA without potential influence of FslE, we compared transport in Schu S4 and a Δ*fupA* Δ*fslE* mutant as well as a mutant complemented with *fupA*. As expected, the ability to transport ^55^Fe at 0.1 *μ*mol/L, lost in the double deletion mutant, was regained upon restoration of *fupA* to the strain (Fig. 7A). We then compared the ability of a 10‐fold excess of metal ions to compete with 0.1 *μ*mol/L ^55^Fe^2+^ uptake in the *fupA* complement. ^55^Fe^2+^ uptake in both Schu S4 and the *fupA* complement was effectively outcompeted by added Fe and Cu, but not by Zn salts (Fig. 7B). This suggested that FupA might specifically facilitate transport of copper in addition to iron.

**Figure 8:**
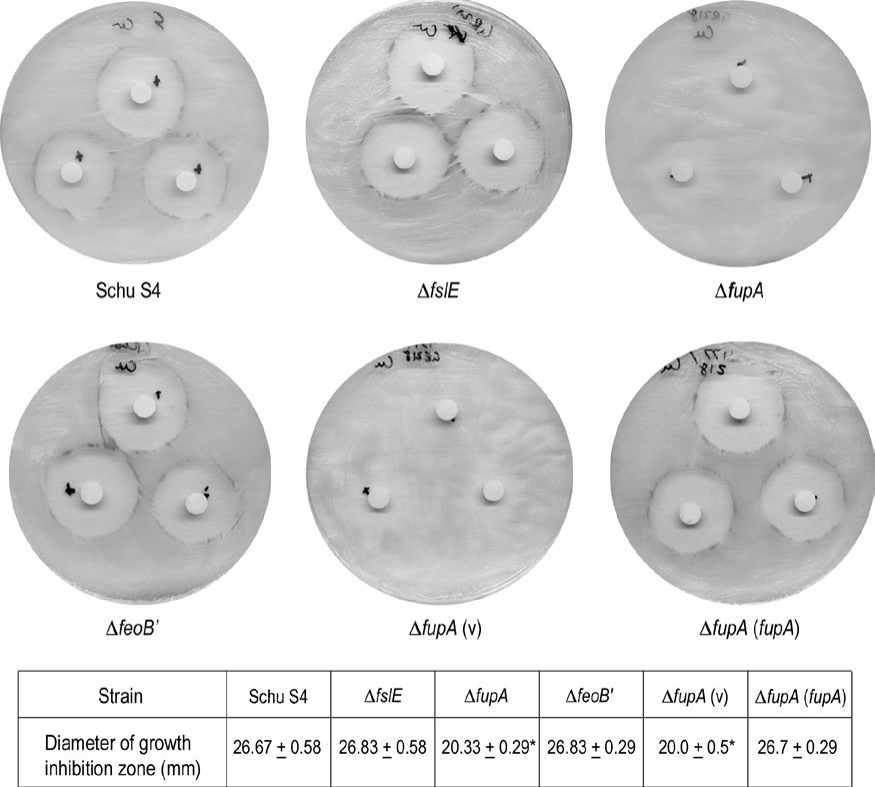
Copper sensitivity of Schu S4 and iron acquisition mutants. Bacteria resuspended in Chamberlin's defined medium (CDM) to an OD_595_ of 1 were spread on Mueller–Hinton agar (MHA)+. 10 *μ*L of 10 mmol/L copper chloride was spotted on paper discs placed on the seeded plates and the effect on growth was evaluated after 48 h. The diameter of growth inhibition was measured and tabulated as mean ± standard error of the mean (SEM). The images document the phenotype and the gray coloration of the growth margins that was absent in the Δ*fupA* mutant.

Copper at low levels is a micronutrient for bacteria, but an excess leads to destruction of iron sulfur clusters of dehydratases in the cell, and is therefore toxic (Macomber and Imlay 2009). We assessed the sensitivity of the different strains to copper using disc diffusion assays. Consistent with a role for FupA in copper transport, the Δ*fupA* mutant specifically showed greater resistance to CuCl_2_ (Fig. 8). Additionally, Schu S4 and the other mutant strains showed a gray coloration at the perimeter of growth suggesting an accumulation of copper sulfide in the cells at the margin of growth. In notable contrast, the Δ*fupA* mutant had a more diffuse growth boundary and showed no gray coloring. Complementation with the *fupA* gene restored the sensitivity to Cu and the gray margin phenotype.

These studies suggested that besides ferrous iron transport, FupA in the outer membrane has additional functions such as transport of copper that may be a micronutrient for *F. tularensis*. FeoB, on the other hand, appears specific for ferrous iron transport.

## Discussion

**Figure 9:**
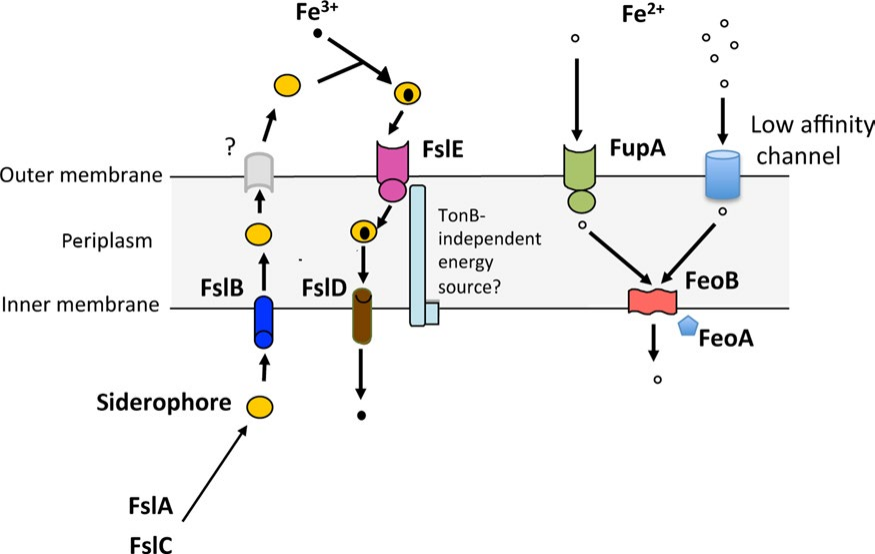
Model for the iron acquisition mechanisms operational in *F. tularensis*. The *fsl* operon encodes cytoplasmic siderophore biosynthetic enzymes FslA and FslC, inner‐membrane proteins FslB and FslD, and outer‐membrane receptor FslE that together effect siderophore‐dependent transport of ferric iron. This system is under Fur control and is induced under iron limitation. The ferrous iron uptake system comprises a high‐affinity transporter FupA and uncharacterized low‐affinity channels in the outer‐membrane and the inner‐membrane transporter FeoB, whose function may be modulated by FeoA. These two systems work as parallel, independent, and mutually compensatory mechanisms to support growth and virulence of the organism.

In the iron‐limiting host environment, high‐affinity acquisition systems for iron are an essential feature of pathogenic organisms. While bacteria in general possess multiple redundant mechanisms for iron acquisition, a limited number of highly specialized strategies may suffice to support the lifestyle adapted to specific niches; *Shigella flexneri* for instance was shown to require only three iron uptake systems to support intracellular growth (Runyen‐Janecky et al. 2003). Reduction in genome size might be expected to limit the number of encoded uptake systems; our studies suggest that the highly virulent *F. tularensis* with a small genome of 1.89 Mb has adapted to support its lifestyle with just two primary mechanisms – the *fsl* system that transports ferric iron complexed to *Francisella* rhizoferrin and the FupA–FeoB pathway for ferrous iron uptake (Fig. 9).

The *fsl* operon encodes five proteins that are conserved across all the closely related strains of *F. tularensis*, and siderophore preparations from *tularensis* and *novicida* strains are identical in structure based on MS‐MS analysis ((Sullivan et al. 2006), data not shown). Our results indicate that the inner‐membrane MFS transporter FslB exports the rhizoferrin synthesized by the enzymes FslA and FslC. The outer‐membrane protein that facilitates siderophore export has not been identified, but our studies suggest that the gene for the protein must lie outside of the *fsl* locus. Our studies further demonstrated that the pathway for uptake of rhizoferrin‐conjugated iron involves the outer‐membrane receptor FslE and the MFS protein FslD that functions as an inner‐membrane channel. These findings are consistent with the observation that FslD shares sequence similarity with the LbtC siderophore transporter in *Legionella pneumophila* (Chatfield et al., 2012). Ferrous iron entry is governed by the high‐affinity transporter FupA in the outer membrane and by FeoB in the inner membrane. The *F. tularensis* genome additionally encodes a FeoA ortholog that may also contribute to FeoB function (Kim et al. 2012; Weaver et al. 2013).

Several lines of evidence demonstrate the limited redundancy of iron acquisition pathways in *F. tularensis*: (1) *fsl* genes for ferric‐siderophore transport and *fupA* and *feoB* for ferrous iron uptake are highly conserved in all *F. tularensis* strains, but neither pathway is essential. Previous studies as well as the current report show that single mutants in the *fupA, feoB,* and *fsl* genes are all viable and virulent, suggesting the existence of compensatory systems to support growth and virulence; the Δ*fsl* mutant demonstrates an increased capacity for ferrous iron uptake and conversely, the siderophore‐mediated uptake pathway is deregulated in Δ*fupA* and Δ*feoB* mutants. (2) We previously demonstrated that a mutant lacking the high‐affinity outer‐membrane transporters FslE and FupA, specific for ferric‐siderophore and ferrous iron, respectively, grew slowly even in iron‐replete conditions (Ramakrishnan et al. 2012). This mutant showed limited intracellular growth in a macrophage cell line. We have shown here that the Δ*feoB* Δ*fslA* mutant that can neither acquire ferrous iron nor synthesize siderophore has an even more severe defect, being completely dependent for growth on exogenously supplied rhizoferrin. This mutant also was unable to grow within mammalian cells. (3) The previously characterized Δ*fupA* Δ*fslE* mutant and the Δ*feoB* Δ*fslA* mutant reported here are both avirulent even when mice were infected with >10,000 × LD_50_ CFUs. These results suggest that no cryptic iron acquisition system is induced upon entry of *F. tularensis* into the host environment.

While levels of ferric and ferrous forms of iron may vary among different tissues within the host, the retention of virulence by the *fsl* and the *feoB* single mutants demonstrates that both ferric and ferrous sources are utilized by the invading pathogen. Ferric iron is the form of iron stored in ferritin or bound to transferrin and lactoferrin while ferrous iron is thought to be the form that is rapidly mobilized and translocated within the cell (Hider and Kong 2013). The *Francisella* siderophore structure appears identical to that of rhizoferrin (Sullivan et al. 2006); with a relatively low affinity for ferric iron (pFe = 19.7) (Carrano et al., 1996a, 1996b), it is not predicted to have the capacity to directly chelate away iron that is more tightly bound to host sources such as transferrin (pFe = 23.6) (Turcot et al. 2000). Rhizoferrin is also produced by isolates of diverse bacteria, both environmental and pathogenic (Münzinger et al. 1999; Burnside et al. 2015). This might suggest that the siderophore is not specialized for use in the mammalian environment but targets iron sources commonly available in multiple niches. The ability of *F. tularensis* to grow on transferrin or heme (Olakanmi et al. 2010; Lindgren et al. 2015) is likely dependent on release of iron from the source by other mechanisms prior to internalization by the *fsl* and/or the *feoB* systems. These uptake systems may also be active within tick vectors, where iron availability after a blood meal is predicted to be abundant.

Siderophore uptake across the outer membrane in Gram‐negative bacteria is typically energized by the proton motive force and is dependent on the interaction of the TonB–ExbB–ExbD complex with the siderophore receptor. However, the *F. tularensis* genome does not encode orthologs of the TonB complex. The FslE and FupA transporters share a high level of sequence similarity, but have different substrate specificity: FslE for the *Francisella* rhizoferrin–iron complex and FupA for ferrous iron and potentially for copper as well. The proteins are also predicted by the PRED‐TMBB program (Bagos et al. 2004a,b) to both fold as beta‐barrels in the outer membrane with periplasmic plug domains similar to TonB‐dependent receptors. The similarity suggests that a common mechanism underlies functioning of the two proteins.

Soluble metal ions like ferrous iron and copper are generally considered to diffuse in through porins in the outer membrane of Gram‐negative bacteria. As of now, functional porin proteins have not been identified in *F. tularensis*. The ability of FupA to function at low concentrations of substrate would suggest that it is involved in an active transport process rather than in general diffusion like the MspA porin of *Mycobacterium smegmatis* which acts as a low‐affinity channel for both iron and copper (Jones and Niederweis 2010; Speer et al. 2013). A TonB‐dependent transporter from the cyanobacterium *Anabaena* with specificity for both iron and copper has been previously reported (Nicolaisen et al. 2010); this protein was thought to transport iron as citrate, but the mechanism was not definitively characterized. Despite the lack of an identifiable TonB complex in *F. tularensis*, it is intriguing to consider that these disparate systems may nevertheless share common mechanistic features.

Like iron, copper at high levels is toxic and one strategy of macrophages for controlling internalized pathogens is to mobilize copper into the phagosome (Hodgkinson and Petris 2012). Following entry into the macrophage, *F. tularensis* passes through the phagosome fairly quickly and is able to suppress innate immune responses, and so it is unlikely to encounter toxic copper levels within the macrophage. In fact, FupA may provide a necessary high‐affinity transport function for copper as it does for iron, nutrients that are normally at limiting levels in the host environment.

The *Francisella* genus is diverse and encompasses human and fish pathogens, tick endosymbionts and environmental isolates that are grouped primarily into two evolutionary clades although isolates representing additional divergent branches of the lineage continue to be identified (Sjödin et al. 2012, 2014; Davenport et al. 2014; Svensson et al. 2015). The *feoB* gene is uniformly present in all branches implying the primary reliance of these organisms on ferrous iron acquisition. The *fsl* siderophore operon and *fupA* are highly conserved in genomes of evolutionary clade I which includes *F. tularensis*, but are less well conserved in clade II. Interestingly, sequence analysis suggests that the siderophore operon encoded in the clade II environmental *Francisella philomiragia* genome as well as the more distantly related *Francisella guangzhouensis* and *Fangia hongkongensis* genomes includes additional biosynthetic genes presumably directing the synthesis of a more complex siderophore than *Francisella* rhizoferrin. Moreover, the siderophore operons in the genomes of clade II fish pathogens *Francisella noatunensis* subsp. *noatunensis* and *F. noatunensis subsp. orientalis* are disrupted. The *fslABCDE*‐encoded *Francisella* rhizoferrin system thus is a feature apparently specific to clade I organisms which, in conjunction with the *fupA* and *feoB*‐mediated ferrous iron uptake, adequately supports the pathogenic lifestyle within the mammalian host and possibly also in the tick vector.

The ability of the Schu Δ*fupA* Δ*fslE* and the Δ*feoB* Δ*fslA* mutants to protect mice from subsequent challenge with virulent bacteria implies that the mutant bacteria survive long enough to generate a robust immune response. It is also possible that they may undergo a very limited replication in vivo since that was found to be important for generation of immunity by other avirulent bacterial mutants (Rockx‐Brouwer et al. 2012). While the mechanisms underlying generation of immunity remain to be determined, these attenuated iron uptake mutants appear to be good candidates for further exploration as live vaccines.

## Methods

### Bacterial strains and media

*F. tularensis* subspecies *tularensis,* Schu S4 (from the Centers for Disease Control and Prevention, Fort Collins, CO), and mutant derivatives were maintained on modified MHA supplemented with horse serum, cysteine, and defined amounts of iron salts including ferrous sulfate (FeSO_4_) and ferric pyrophosphate (FePPi). *F. tularensis* strains were grown in liquid CDM (Chamberlain 1965) or in tryptic soy broth supplemented with 0.1% cysteine (TSB/c) at 37°C with shaking. Bacterial culture optical densities were determined at 595 nm (OD_595_) using a microplate reader (iMark, Bio‐Rad Laboratories, Hercules, CA, USA). For growth determinations in defined media, bacterial cultures were grown initially in iron‐replete CDM overnight. Cultures were then washed in chelex treated CDM three times and inoculated into che‐CDM supplemented with known quantities of FePPi (2.5 and 0.125 mg/L) and FeSO_4_ (2 mg/L and 0.2 mg/L). Bacteria in the exponential growth stage were inoculated to an OD_595_ of 0.01 in the respective growth media and grown at 37°C with shaking for 24 h. The Schu Δ*feoB*′ Δ*fslA* mutant was maintained on MHA agar supplemented with siderophore purified from LVS (as described in Pérez and Ramakrishnan 2014).

### Generation of Schu S4 iron acquisition mutants and complementation

Marker less deletion mutants were generated by a two‐step process using suicide plasmids with 5′ and 3′ flanking sequences as previously described (Sullivan et al. 2006; Ramakrishnan et al. 2008). Δ*fslA* and Δ*feoB*′ mutants in the Schu S4 background were obtained as described previously for LVS (Sullivan et al. 2006; Pérez and Ramakrishnan 2014). Generation of the Δ*fslF* mutant and deletion of the entire *fslABCDEF* locus (Δ*fsl*) in Schu S4 and LVS backgrounds was similarly accomplished using primers listed in (Table S1). The suicide plasmids were introduced into Schu S4 or LVS by electroporation and integrants were selected on MHA+ containing 15 *μ*g/mL kanamycin. Mutants were obtained by selection on sucrose containing MHA+, screened for loss of the kanamycin resistance marker and confirmed by PCR. The Schu Δ*fslA* Δ*feoB*′ strain required supplementation with purified *F. tularensis* siderophore for growth, as previously seen with the LVS Δ*fslA* Δ*feoB*′ mutant (Pérez and Ramakrishnan 2014).

#### Complementation

Plasmids for complementation in *cis* or in *trans* were introduced by electroporation and selected on media containing kanamycin

#### p*feoB*

Both the Δ*feoB*′ and double deletion mutants were complemented in *cis* with a wild‐type copy of *feoB* present on an integrating suicide plasmid, p463_*feoB* previously described for the complementation of the LVS *feoB*′ mutants (Pérez and Ramakrishnan 2014). Complemented mutants were selected with kanamycin and confirmed by PCR from DNA purified from the bacteria.

#### p*fslAB,* p*fslAC,* and p*fslABC*

The LVS *Δfsl* mutant was complemented in *trans* to assess *fsl* genes required for siderophore production. Complementation plasmids were derived from the plasmid pGIR458 (Sullivan et al. 2006) that contains a wild‐type copy of Schu S4 *fslA* with its native promoter. To generate p*fslAC,* the *fslC* gene was amplified from Schu S4 genomic DNA with primers 5′ctactggagctcTTAAATCATCTAATTTTAAAAATAAGG 3′ and 5′ ctactgggatccTTATTGATGTGTTTGTCTAACTC 3′ and then cloned downstream of *fslA* in pGIR458 at *Sac*I and *Bam*HI restriction enzyme sites. The plasmid p*fslAB* was generated by the amplification of *fslB* containing sequences with primers 5′‐ctactggcggccgcTGTTAAATGCAAATCCTGTCG 3′ and 5′‐ctactggagctcCTATTTAGACATTTATTAATTCC 3′ and cloning into pGIR458 at the NotI and *Sac*I restriction enzyme sites. The plasmid p*fslABC* was generated with the amplification of *fslBC* with primers 5′‐ctactggcggccgcTGTTAAATGCAAATCCTGTCG 3′ and 5′‐ctactgggatccTTATTGATGTGTTTGTCTAACTC 3′ and ligation into pGIR458 at NotI and *Bam*HI restriction enzyme sites. The parent plasmid pFNLTP6 (V) (Maier et al. 2004) was used as a negative control in our studies.

#### p*fslE*, p*fslDE,* and p*fslDEF*

The Schu Δ*fsl* mutant was complemented in *cis* to identify *fsl* genes involved in siderophore‐mediated iron uptake. The p*fslE* plasmid pGIR474 that has the *fslE* gene under control of its native *fslA* promoter in the integrative plasmid pGIR459 has been previously described (Sen et al. 2010). The forward primer 5′‐ctactgtccggaCTCAGAGTTAGACAAACACAT 3′ with reverse primers 5′‐ctactggcggccgcTTAAAGATATACAGCCATATCT 3′ and 5′‐ctactggcggccgcGCTCTATAGCAATATCACCAAC 3′ were used to amplify *fslDE* and *fslDEF* sequences, respectively, from Schu S4 chromosomal DNA. The sequences were cloned under control of the *fslA* promoter in pGIR459 to generate p*fslDE* and p*fslDEF,* respectively. The parent plasmid pGIR459 was used as vector control for complementation studies.

### *F. tularensis* siderophore detection with the Chrome Azurol S (CAS) assay

As previously described in Pérez and Ramakrishnan (2014), an adaptation of the liquid CAS assay was used to determine the presence of the *F. tularensis* siderophore under iron limitation. Bacterial cultures were grown in iron replete or iron‐limiting FePPi (2.5 and 0.125 *μ*g/mL) supplemented che‐CDM overnight at 37°C with shaking. Cultures were centrifuged at 9,0000*g* for 5 min and supernatants were collected and added to equal parts CAS solution (100 *μ*L) and 2 *μ*L of the shuttle solution in a 96‐well plate. The CAS activity was normalized to cell density

### Growth on agar plates

To assess the growth of Schu S4 and Schu S4 iron acquisition mutants on agar, overnight cultures were washed in che‐CDM and 5 *μ*L of 10‐fold serially diluted suspensions were spotted on modified Muller Hinton agar plates supplemented with FePPi or FeSO_4_ or on CDM plates without iron.

### Disc diffusion test

Overnight cultures grown to logarithmic phase in TSB/c were resuspended to OD 1 in CDM. The cells were uniformly plated on MHA using 100 *μ*L of each of the OD 1 cultures. The plates were dried at room temperature for 15 min. Sterile 6‐mm paper discs were placed on the plates and 5 *μ*L of the test solutions were spotted. All experiments were carried out in triplicate. After drying, the plates were incubated at 37°C for 2 days. The effect on growth was evaluated by measuring the diameter of the inhibition zone.

### ^55^Fe uptake assays

^55^Fe uptake with ^55^FeCl_3_ (PerkinElmer Life Sciences; 21.95 mCi/mg, 38.59 mCi/mL) was studied using previously described protocols (Ramakrishnan et al. 2012; Ramakrishnan and Sen 2014). Prior to ^55^Fe uptake assays, bacterial strains (except for the Δ*feoB*′ Δ*fslA* mutant) were grown overnight in low iron liquid che‐CDM at 37°C with shaking. For experiments involving the Δ*feoB*′ Δ*fslA* mutant, the double mutant bacteria were scraped off after growth on MHA+ plates supplemented with siderophore on the day of the experiment and incubated in che‐CDM without iron for 3 h at 37°C with shaking. Control cultures grown in low iron che‐CDM were washed with fresh che‐CDM and similarly incubated in che‐CDM. Bacterial cells were brought to an OD_595_ of 0.2 in che‐CDM, and 0.1 mL of each bacterial suspension was added to wells of 96‐well filter plates (Millipore, Billerica, MA, USA) containing 90 *μ*L of che‐CDM. After a 10‐min incubation at 37°C, uptake assays were initiated by the addition of 10 *μ*L of ^55^Fe labeling mix to the bacteria in the filter wells. ^55^Fe^3+^‐siderophore uptake reactions were carried out in the presence of 10 mmol/L citrate. Siderophore complexes were prepared by incubating 30 *μ*mol/L ^55^FeCl_3_ with 100 *μ*mol/L siderophore for 30–60 min at room temperature. The uptake reaction contained 1.5 *μ*mol/L ^55^Fe in complex with siderophore. For ferrous iron uptake studies, the final transport assay contained ^55^FeCl_3_ at concentrations of 0.1 *μ*mol/L (high‐affinity transport) or 3 *μ*mol/L (low‐affinity uptake) in the presence of 5 mmol/L ascorbate to keep the iron reduced. ^55^Fe accumulation was assessed at 5 and 10 min by scintillation counting of filtered cells. Bacterial protein concentration was analyzed by the Pierce BCA assay (Thermo‐Fisher Scientific, Middletown, VA, USA). All strains were further tested in either triplicate or quadruplicate and rates of transport were normalized to protein concentration (pmol/min/mg).

### Intracellular replication

Intracellular replication of Schu S4 and mutants was assessed in murine macrophage‐like cells J774A.1 (ATCC TIB‐67) as previously described (Sen et al. 2010; Pérez and Ramakrishnan 2014). J774A.1 cells were maintained in high glucose Dulbecco's modified Eagle's medium (DMEM) supplemented with 10% FBS at 37°C with 5% CO_2_ and split 1:10 per passage. Cells were counted on an automated cell counter (TC10, Bio‐Rad Corporation, Hercules, CA, USA) and seeded at a concentration of 2 × 10^5^ cells per well in 24‐well plates the day before the assay. Bacteria were added at a multiplicity of infection (MOI) of 10 into four wells per group. HepG2 cells also used to assess replication were maintained in DMEM supplemented with 10% FBS, 5% glutamate, and grown at 37°C with 5% CO_2_ and split 1:10 per passage. Cells were seeded at a concentration of 2 x 10^5^ per well in a 24‐well plate the day before the assay. Bacteria were added at an MOI of 100 into four wells per group.

### Mouse infection studies

All animal protocols were approved by the Animal Care and Use Committee (ACUC) of the University of Virginia, and the vivarium is accredited by the Association for Assessment Accreditation of Laboratory Animal Care International. Mice were anesthetized with a cocktail of ketamine‐HCl‐xylazine. Previously titered frozen bacterial cultures were thawed to room temperature and diluted in 0.9% sterile saline solution to 250 CFU/mL and 100 *μ*L (25 CFU) aliquots were subcutaneously (S.C) injected into 4–6 week old C57BL/6 male mice (five mice per group) (Jackson laboratories, Bar Harbor, ME) (Ramakrishnan et al. 2012). CFUs were determined after plating bacterial dilutions on MHA plates. MHA topically supplemented with *F. tularensis* siderophore was used to determine CFUs of the double deletion mutant. Clinical scores were determined for mice over the course of infection and mice were euthanized at a humane endpoint if symptoms of irreversible morbidity were observed. Survivors were subsequently challenged by S.C. delivery of 25 CFU of wild‐type Schu S4 and monitored over 21 days.

LD_50_ studies: Three groups of male C57BL/6 (4–6 weeks old) mice were S.C. injected with 0.1‐mL saline solution containing either 14,700 CFU, 3000 CFU, or 173 CFU of the Δ*feoB*′Δ*fslA* mutant. CFUs were determined by plating bacterial dilutions on MHA plates supplemented with *F. tularensis* siderophore. Mice were monitored for 21 days and then challenged with 33 CFU of wild‐type Schu S4. CFUs were determined by plating on MHA plates.

### Statistical analysis

Prism 4.0 (GraphPad Software, Inc., San Diego, CA) was used for analysis of data. Statistical comparison of values was accomplished using *t* test function and the log‐rank test function was used to evaluate mouse survival curves.

## Conflict of Interest

None declared.

## Supporting information


**Table S1.**Primers used in the study.
