# Supplementary material for: Two parallel pathways for ferric and ferrous iron acquisition support growth and virulence of the intracellular pathogen Francisella tularensis Schu S4
Source: Microbiologyopen. 2016 Feb 25;5(3):453–68. doi: 10.1002/mbo3.342 (PMC4905997; doi:10.1002/mbo3.342)
Supplement: Supplementary file 1 — Table S1.Primers used in the study. [file MBO3-5-453-s001.docx]

Table S1. Primers used in the study

| Primer | Purpose |
| --- | --- |
| Generation of Δ*fsl* mutants  5’ ctactggctagcTTTGTGAAACAACCTTACCAACC 3’  5’ ctactgtccggaTCAGAAAAGCTGTTGTGAGATTG 3’  5’ ctactgtccggaGTTGGTGATATTGCTATAGAGC 3**'**  5**'** ctactggcggccgcATGCTAAGAAAGTGATAG 3**'**  Generation of Δ*fslF* mutant  5**'** ctactggctagcTCACCATTTCCTAAAATGATT 3**'**  5**'** ctactgtccggaCTTAAAGATATACAGCCATATC 3**'**  5’ ctactgtccggaGTTGGTGATATTGCTATAGAGC 3**'**  5**'** ctactggcggccgcATGCTAAGAAAGTGATAG 3**'**  Complementation with *feoB+*  5’ ctactgtccggaGCCAATCCAAGATATGGTG 3’  5’ ctactgcatatgATTCAAATTAGAATTTTAAGAGC 3’  Complementation with *fslAB*  5’ ctactggcggccgcTGTTAAATGCAAATCCTGTCG 3’  5’ ctactggagctcCTATTTAGACATTTATTAATTCC 3’  Complementation with *fslAC*  5’ctactggagctcTTAAATCATCTAATTTTAAAAATAAGG 3’  5’ ctactgggatccTTATTGATGTGTTTGTCTAACTC 3’  Complementation with *fslABC*  5’ ctactggcggccgcTGTTAAATGCAAATCCTGTCG 3’  5’ ctactgggatccTTATTGATGTGTTTGTCTAACTC 3’ | Forward primer for 5’ flank  Reverse primer for 5’ flank  Forward primer for 3’ flank  Reverse primer for 3’ flank  Forward primer for 5’ flank  Reverse primer for 5’ flank  Forward primer for 3’ flank  Reverse primer for 3’ flank  Forward primer for 5’ flank  Reverse primer for 3’ flank  Forward primer for 5’ flank  Reverse primer for 3’ flank  Forward primer for 5’ flank  Reverse primer for 3’ flank  Forward primer for 5’ flank  Reverse primer for 3’ flank |
